# Cohort Profile: the Etude Epidémiologique sur les Petits Ages Gestationnels-2 (EPIPAGE-2) preterm birth cohort

**DOI:** 10.1093/ije/dyaa282

**Published:** 2021-06-24

**Authors:** Elsa Lorthe, Valérie Benhammou, Laetitia Marchand-Martin, Véronique Pierrat, Cécile Lebeaux, Mélanie Durox, François Goffinet, Monique Kaminski, Pierre-Yves Ancel, the EPIPAGE-2 Study group, D Astruc, P Kuhn, B Langer, J Matis, C Ramousset, X Hernandorena, P Chabanier, L Joly-Pedespan, M Rebola, M J Costedoat, A Leguen, C Martin, B Lecomte, D Lemery, F Vendittelli, E Rochette, G Beucher, M Dreyfus, B Guillois, Y Toure, D Rots, A Burguet, S Couvreur, J B Gouyon, P Sagot, N Colas, A Franzin, J Sizun, A Beuchée, P Pladys, F Rouget, R P Dupuy, D Soupre, F Charlot, S Roudaut, A Favreau, E Saliba, L Reboul, E Aoustin, N Bednarek, P Morville, V Verrière, G Thiriez, C Balamou, C Ratajczak, L Marpeau, S Marret, C Barbier, N Mestre, G Kayem, X Durrmeyer, M Granier, A Lapillonne, M Ayoubi, O Baud, B Carbonne, L Foix L’Hélias, F Goffinet, P H Jarreau, D Mitanchez, P Boileau, C Duffaut, E Lorthe, L Cornu, R Moras, D Salomon, S Medjahed, K Ahmed, P Boulot, G Cambonie, H Daudé, A Badessi, N Tsaoussis, M Poujol, A Bédu, F Mons, C Bahans, M H Binet, J Fresson, J M Hascoët, A Milton, O Morel, R Vieux, L Hilpert, C Alberge, C Arnaud, C Vayssière, M Baron, M L Charkaluk, V Pierrat, D Subtil, P Truffert, S Akowanou, D Roche, M Thibaut, C D’Ercole, C Gire, U Simeoni, A Bongain, M Deschamps, M Zahed, B Branger, J C Rozé, N Winer, G Gascoin, L Sentilhes, V Rouger, C Dupont, H Martin, J Gondry, G Krim, B Baby, I Popov, M Debeir, O Claris, J C Picaud, S Rubio-Gurung, C Cans, A Ego, T Debillon, H Patural, A Rannaud, E Janky, A Poulichet, J M Rosenthal, E Coliné, C Cabrera, A Favre, N Joly, A Stouvenel, S Châlons, J Pignol, P L Laurence, V Lochelongue, P Y Robillard, S Samperiz, D Ramful, P Y Ancel, H Asadullah, V Benhammou, B Blondel, M Bonet, A Brinis, M L Charkaluk, A Coquelin, V Delormel, M Durox, S Esmiol, M Fériaud, L Foix-L’Hélias, F Goffinet, M Kaminski, G Kayem, K Khemache, B Khoshnood, C Lebeaux, E Lorthe, L Marchand-Martin, L Onestas, V Pierrat, M Quere, J Rousseau, A Rtimi, M J Saurel-Cubizolles, D Tran, D Sylla, L Vasante-Annamale, J Zeitlin

**Affiliations:** 1 Université de Paris, Epidemiology and Statistics Research Center/CRESS, INSERM (U1153 - Obstetrical, Perinatal and Pediatric Epidemiology Research Team [EPOPé]), INRA, F-75004 Paris, France; 2 EPIUnit—Institute of Public Health, University of Porto, Porto, Portugal; 3 CHU Lille, Department of Neonatal Medicine, Jeanne de Flandre Hospital, Lille, France; 4 Neonatal Intensive Care Unit, Centre Hospitalier Intercommunal de Créteil, Ile de France, France; 5 Reseau Perinatal, Val de Marne, Ile-de-France, France; 6 Maternité Port-Royal, AP-HP, APHP.Centre - Université de Paris, FHU PREMA, Paris, France; 7 Clinical Research Unit, Center for Clinical Investigation P1419, APHP.CUP, F-75014, Paris, France

## Why was the EPIPAGE-2 cohort set up?

Prematurity has shown an upward trend since 1990, accounting for about 10% of births worldwide, representing almost 15 million babies born every year before 37 weeks’ gestation.^1^^,^^2^ In France, the preterm birth rate was 7.4% in 2010, with about 60 000 babies born preterm every year.^3^

The burden of preterm birth is substantial: it remains a major cause of child mortality during both the neonatal period and childhood before the age of 5 years.^4^ Among survivors, the frequency of prematurity-related health problems and developmental deficiencies is substantial, in the short and in the long term after birth.^2^^,^^5^ With prematurity and survival rates both increasing, these ‘individuals born preterm’ represent a growing share of the population, displaying specific health care and support needs.

Population-based cohort studies are the methodology of reference for assessing the longitudinal evolution of these fragile infants. Several European and international cohorts have been conducted since the late 1990s, mainly focusing on children born extremely preterm, between 22 and 26 weeks’ gestation.^6–9^ Only a few studies included infants born very (27–31 weeks) or moderately (32–34 weeks) preterm, although they are more numerous and with a greater impact on public health indicators.^10^^,^^11^

The first EPIPAGE (Etude Épidémiologique sur les Petits Âges Gestationnels) cohort study was launched in 1997 in nine French regions, including births occurring at 22–32 weeks’ gestation, with follow-up steps until age 8 years.^12^ The cohort provided estimates of mortality, morbidity and disability and health care needs and greatly contributed to changing practices in the neonatal period and after hospital discharge.^13^^,^^14^

Medical practices and the organization of care vary widely across countries and have markedly evolved over the past two decades.^15–17^ The prognosis of very preterm infants has changed accordingly, raising new questions and requiring new assessments.^17^ We therefore set up the EPIPAGE-2 cohort, a new longitudinal study of preterm infants, with the following objectives: to provide actualized estimates of short- and long-term outcomes for extremely, very and moderately preterm babies and their families; to study changes in practices at both individual and organizational levels and their impact on child health and development; and to explore aetiologies of preterm birth and identify early predictors of adverse outcomes.^17^

**Figure 1:**
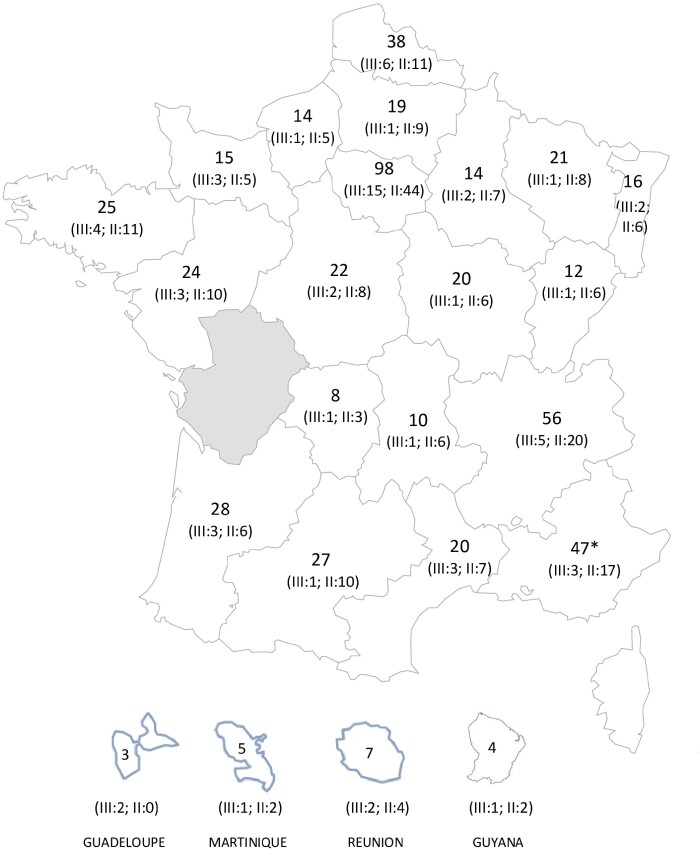
Number of maternity units in the French regions involved in the EPIPAGE-2 cohort study. III, type III maternity unit; II, type II maternity unit. *Including maternity units from Corsica

EPIPAGE-2 is a population-based cohort study, set up in 2011 in 25 regions in France (21 of the 22 metropolitan regions and four overseas regions). Only one region (Poitou-Charentes), accounting for 2.2% of all births in France in 2011, did not participate because of organizational issues. All maternity units and neonatology departments participated in the recruitment (Figure 1).

The establishment of the cohort required a close collaboration between the EPOPé research team [National Institute of Health and Medical Research (Inserm U1153), Université de Paris] on the one hand, and a national group of participants from 555 French maternity units, 281 neonatology departments and 39 perinatal networks and parent associations, on the other hand. At a national level, a steering committee, including epidemiologists, paediatricians and obstetricians, was in charge of designing the scientific project and the overall study organization. In each region, a coordinating committee was responsible for implementing the study at the regional level. Local clinical teams from maternity and neonatal units were involved in inclusions and data collection, with the help of regional coordinators.

The study relies on several sources of funding, including support from the French National Institute of Public Health Research (IRESP TGIR 2009–01 programme)/Institute of Public Health and its partners [the French Health Ministry, the National Institute of Health and Medical Research (INSERM), the National Institute of Cancer, the National Solidarity Fund for Autonomy (CNSA)], the National Research Agency through the French EQUIPEX program of investments for the future (grant no. ANR-11-EQPX-0038) and the PremUp Foundation. Additional funding was obtained from the Fondation pour la Recherche Medicale (SPF 20160936356) and Fondation de France (00050329 and R18202KK [Grand Prix]).

As required by French law and regulations, recruitment and follow-ups were approved by the national data protection authority (Commission Nationale de l’Informatique et des Libertés, CNIL DR-2011–089, DR-2012–246, DR-2013–406, DR-2016–290) and by the appropriate ethics committees, i.e. the advisory committee on the treatment of personal health data for research purposes (Comité Consultatif sur le Traitement de l'Information en matière de Recherche, CCTIRS, reference nos. 10–626, 12–109 and 16–263) and the committee for the protection of people participating in biomedical research (Comité de Protection des Personnes, CPP, reference nos. 2011-A00159-32 and 2016-A0033-48).

## Who is in the EPIPAGE-2 cohort?

Recruitment took place in all maternity units of the 25 participating regions during an 8-month period for extremely preterm births (22–26 weeks) and during a 6-month period for very preterm births (27–31 weeks) (Figure 1). A sample of moderately preterm births (32–34 weeks) was recruited during a 5-week period. Further details can be found in the study protocol.^17^

Eligibility was based on gestational age at birth. Participation in the study was proposed to the parents of all eligible children after they received appropriate information, in the maternity or neonatal unit. During recruitment, regional coordinators visited all maternity units to ensure the identification of all eligible children. Only families who orally agreed to participate were included. The only exclusion criterion was refusal to participate.

During the recruitment period, 8400 births were eligible, including terminations of pregnancy, stillbirths and live births, among whom 7804 (93%) were enrolled in the study. Refusal rate at baseline was 7% (*n* = 596). With ethics committee approval, a small number of basic perinatal data were collected from birth certificates for all eligible births, in order to characterize non-participants. Neonates whose parents refused participation were more frequently born at 32–34 weeks’ gestation and to younger mothers of lower socioeconomic position (SEP) (Table 1).

**Table 1:** Comparison of participants and non-participants at recruitment and at follow-up invitation

	No. of events/No. in group %^a^										
	Recruitment (*N* = 8400)					Invitation to follow-up among survivors at discharge (*N* = 4467)					
	Participants *n* = 7804		Refusals *n* = 596		*P*-value	Participants *n* = 4312			Refusals *n* = 155		*P*-value
Maternal characteristics											
Age, years											
<20	269/7781	2.9	47/596	8.4	<0.001		138/4312	2.5	10/155	7.8	0.009
20-35	6103/7781	79.4	435/596	71.6			3424/4312	80.3	118/155	74.8	
>35	1409/7781	17.7	114/596	20.0			750/4312	17.2	27/155	17.4	
Parents’ socioeconomic position^b^											
Manager	1386/6965	21.6	35/447	8.6	<0.001		909/4090	23.2	14/133	11.0	<0.001
Professional	1393/6965	21.1	62/447	12.8			873/4090	22.1	11/133	11.5	
Intermediate^c^	1850/6965	26.8	93/447	23.1			1118/4090	27.3	35/133	30.1	
Sales and services worker	1004/6965	14.0	69/447	12.5			583/4090	13.8	24/133	18.7	
Manual worker	934/6965	12.1	87/447	18.3			482/4090	10.9	32/133	17.1	
Unknown	398/6965	4.4	101/447	24.7			125/4090	2.7	17/133	11.6	
Smoking during pregnancy, yes	1534/7457	20.3	113/552	23.8	ns		896/4166	20.4	38/147	26.8	ns
Obstetric characteristics											
No previous pregnancy	2748/7787	36.3	203/590	33.4	ns		1561/4303	37.1	52/155	40.8	ns
Multiple pregnancy	1987/7804	30.5	109/596	16.5	<0.001		1451/4312	35.7	51/155	31.5	ns
Neonatal characteristics Gestational age at birth, weeks											
22-26	3045/7804	18.4	215/596	13.8	<0.001		529/4312	4.4	22/155	4.1	<0.001
27-31	3510/7804	28.7	235/596	20.4			2648/4312	29.6	75/155	19.1	
32-34	1249/7804	52.9	146/596	65.8			1135/4312	66.0	58/155	76.8	

ns, non significant.

a Weighted percentages.

b Defined as the highest occupational status between current (or former) occupations of the mother and the father, or mother only if living alone, and based on the Classification of Professions and Socioprofessional Categories, developed by the French National Institute of Statistics and Economic Studies.

c Intermediate socioeconomic position includes employees from administration and public service, self-employed and students.

The follow-up was proposed to all families of children discharged alive from hospital (i.e. 4467 children). The families of 155 children (3%) had agreed to participate at baseline but secondarily refused to take part in the follow-up. Thus, 4312 children were eligible for the follow-up. Families who refused the follow-up had a similar profile to that described for the initial refusals in terms of maternal age, SEP and gestational age at birth (Table 1). All children whose parents agreed to participate in the follow-up were invited at each follow-up step, whether they had participated in the previous follow-up or not, unless parents asked to stop their participation in the study.

## How often have they been followed up?

**Figure 2:**
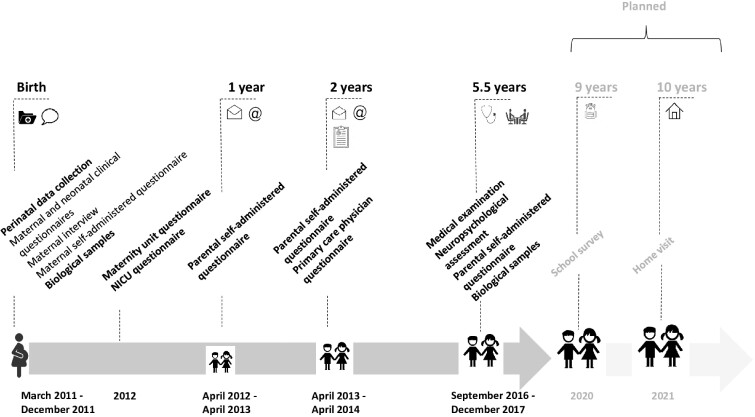
Assessments and data collection. NICU, neonatal intensive care unit

From 2011 to 2017, evaluation at baseline and three follow-up steps were performed at 1 and 2 years’ corrected age and at 5.5 years (Figure 2).

### Assessment schedule

At birth and during the neonatal period, maternal and neonatal data were extracted from medical records. Moreover, we interviewed mothers in the neonatal units during the infant’s hospitalization and mothers completed a self-administered questionnaire just before the baby’s discharge.

At each follow-up step, parents completed self-administered questionnaires. Additionally, at 2 years’ corrected age, the child’s referring physician completed a standardized questionnaire. At 5.5 years, children had a clinical examination by a physician and a cognitive assessment by a neuropsychologist, both performed in one of 110 dedicated examination centres in all participating regions. All professionals were specifically trained to ensure homogeneity in data collection.

### Follow-up perspectives

To better understand the specific educational difficulties encountered by very preterm children, a school survey will be performed in September 2020, when most children will be in the 4th year of primary school. The survey will comprise tests in French and arithmetic and a few questions for children about their well-being at school. A questionnaire will be completed by the teacher on the child’s behaviour and position in the class.

Finally, children will be directly interviewed for the first time at 10.5 years of age (2021–22), at home. This step of follow-up will allow for assessing development and health status and collecting biological samples

### What is attrition like?

**Figure 3:**
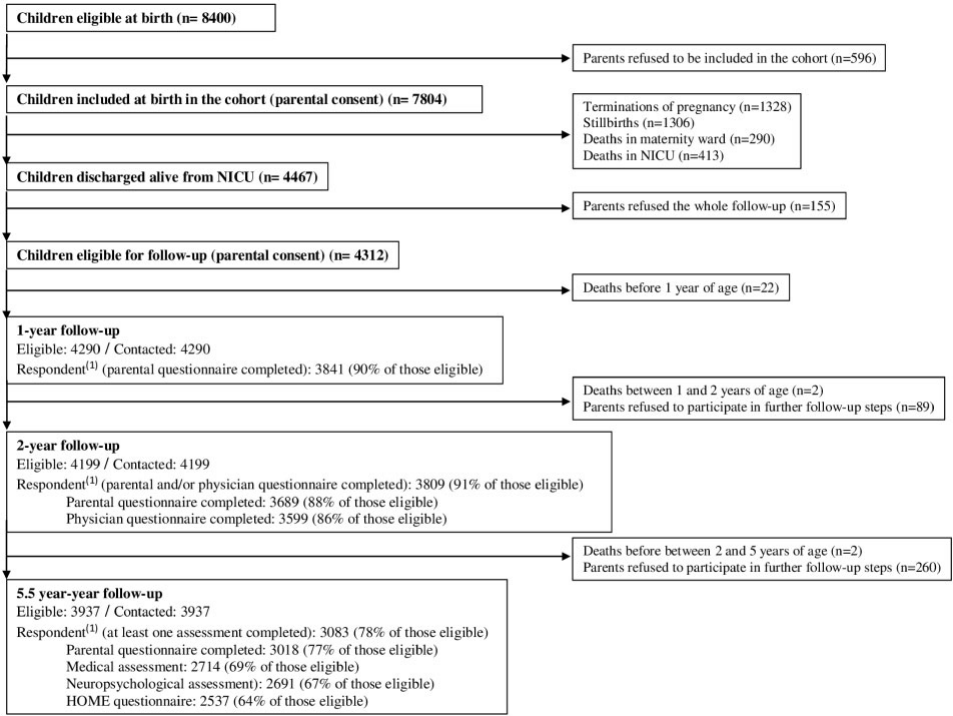
Participation from birth to 5.5 years in the EPIPAGE-2 cohort. (1) Respondent: includes complete and incomplete questionnaires. No completed questionnaire whatever the follow-up step: 117/4286 (3%). NICU, neonatal intensive care unit

Overall, the families extensively collaborated in the study, with a participation rate of 93% (7804 children) at baseline. A total of 26 children died between their discharge from hospital and the 5.5-year follow-up. The families of 504 children (11%) decided to stop their participation in follow-up: 155 (3%) before 1 year, 89 (2%) between 1 and 2 years and 260 (6%) at 5.5 years. At 5.5 years, 3937 (86%) children discharged alive from neonatal units remained in the cohort (Figure 3).

The parents’ response rates were 90% and 91% at 1 and 2 years, respectively. At 5.5 years, at least one assessment was performed for 3083 (78%) children (Figure 3). A total of 117 (3%) children, still alive, were never assessed whatever the follow-up step, despite parents never declining participation in the study.

Mothers of children who did not participate at 5.5 years were younger, had lower educational level and SEP and were more frequently single than those who did participate; however, the two groups did not differ in children’s characteristics (Table 2).

**Table 2:** Comparison of respondents and non-respondents at 5.5 years among the 4286 eligible children

	No. of events/No. in group %^a^				
	Respondent at 5.5 years		Non-respondent at 5.5 years		
	*n *= 3083		*n* = 1203		*P*-value
Gestational age, weeks					
24-26	379/3083	4.5	143/1203	4.0	0.0003
27-31	1934/3083	31.1	701/1203	26.2	
32-34	770/3083	64.4	359/1203	69.8	
**Maternal characteristics at birth**					
Maternal age at birth, years					
<20	67/3083	1.4	70/1203	5.0	<0 .001
20-35	2476/3083	80.8	929/1203	79.4	
>35	540/3083	17.8	204/1203	15.6	
Mother born in France	2506/3074	84.4	822/1175	72.2	<0 .001
Mother living with a partner	2725/2925	94.1	977/1130	85.8	<0 .001
Parents’ socioeconomic position^b^					
Manager	750/2959	26.4	157/1108	15.8	<0 .001
Professional	704/2959	24.8	168/1108	15.9	
Intermediate^c^	766/2959	25.4	349/1108	32.3	
Sales and services worker	370/2959	12.0	207/1108	17.5	
Manual worker	315/2959	9.6	157/1108	13.3	
Unknown	54/2959	1.7	70/1108	5.1	
Maternal level of education					
Lower secondary	845/2982	26.9	472/1070	42.2	<0 .001
Upper secondary	616/2982	20.5	258/1070	24.4	
Post-secondary, not tertiary	629/2982	21.6	158/1070	14.5	
Bachelor degree or more	892/2982	31.0	182/1070	18.9	
Multiple pregnancy	1079/3083	37.4	366/1203	32.0	0.001
**Children characteristics**					
Male	1638/3083	54.9	621/1203	49.5	0.02
Small-for-gestational age^d^	1069/3082	34.1	407/1203	33.5	ns
Severe neonatal morbidities^e^	376/2936	7.0	140/1130	5.9	ns
Cerebral palsy at 2 years	104/2848	2.4	33/750	2.0	ns

ns: non significant.

a Weighted percentages.

b Defined as the highest occupational status between current (or former) occupations of the mother and the father, or mother only if living alone, and based on the Classification of Professions and Socioprofessional Categories, developed by the French National Institute of Statistics and Economic Studies.

c Intermediate socioeconomic position includes employees from administration and public service, self-employed and students.

d Defined as birthweight less than the 10th percentile for gestational age and sex based on French intrauterine growth curves (Ego 2016).

e Defined as severe bronchopulmonary dysplasia or necrotizing enterocolitis stage 2–3 or severe retinopathy of prematurity stage >3 or any of the following severe cerebral abnormalities on cranial ultrasonography: intraventricular haemorrhage grade III or IV or cystic periventricular leukomalacia.

## What has been measured?

Overall, almost 5 000 variables have been collected from baseline to 5.5-year follow-up. All questionnaires are available at [https://pandora-epipage2.inserm.fr/public/index.php]. Table 3 summarizes the main types of data collected on maternal health, antenatal management, parental sociodemographic characteristics and family lifestyle. Table 4 presents the data collected on child’s health, development and health care use.^18–20^ The standardized scales used in the EPIPAGE-2 questionnaires are presented in Table 5.^21–32^

**Table 3:** Data collected on maternal health, antenatal management, family’s sociodemographic characteristics and lifestyle

	Birth	1 year	2 years	5.5 years
**Maternal health**				
Medical history	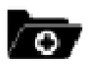	–	–	–
Obstetric history	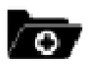	–	–	–
Pregnancy complications	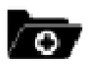	–	–	–
Post-partum depression	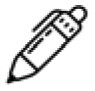	**-**	**-**	**-**
Post-partum anxiety	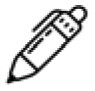	–	–	–
Global self-rated health	–	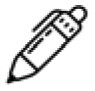	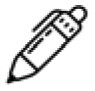	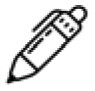
Mental self-rated health	–	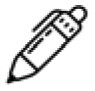	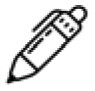	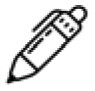
Physical self-rated health	–	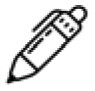	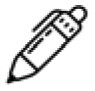	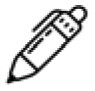
**Antenatal management**				
Diagnosis and medical management	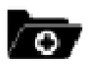	–	–	–
Ultrasonography and blood tests	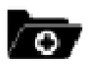	–	–	–
Treatments and medications	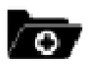	–	–	–
Hospitalizations during pregnancy	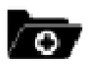	–	–	–
Indications for medical interventions	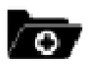	–	–	–
Delivery and post-partum	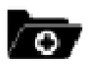	–	–	–
**Parental sociodemographic characteristics**				
Familial status		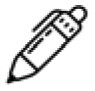	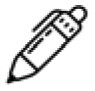	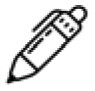
Occupational status	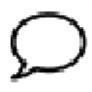	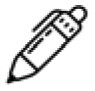	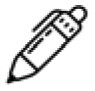	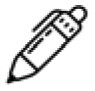
Educational level	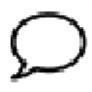	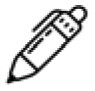		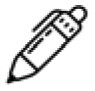
Country of birth/nationality	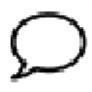	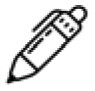		
**Family’s lifestyle, living conditions and living standards**				
Household composition	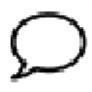	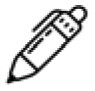	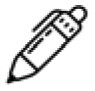	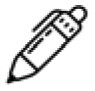
Monthly household income	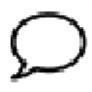	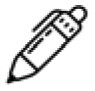		
Social security coverage	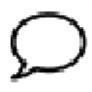	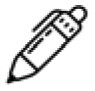		
Type of housing	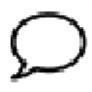	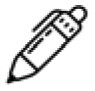		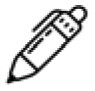
Language spoken at home	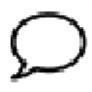	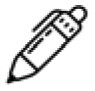		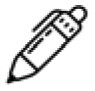

The table specifies whether the information was collected from medical records (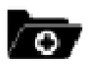), mother’s interview (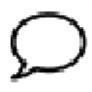), parental self-administered questionnaire (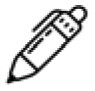) or not collected at this follow-up (-).

**Table 4:** Data collected on child’s health, development, health care use

	Birth	1 year	2 years	5.5 years
Gestational age^a^	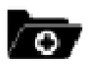	–	–	–
Sex	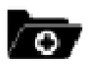	–	–	–
Health and growth				
Apgar score	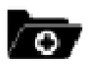	–	–	–
Anthropometric measures	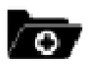	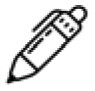	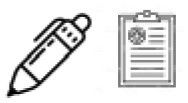	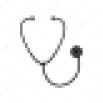
Blood pressure	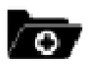	–	–	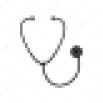
Cardiovascular diseases	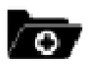	–	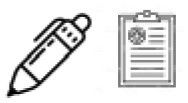	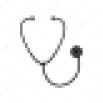
Respiratory diseases	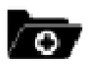	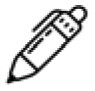	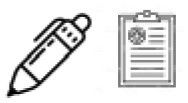	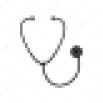
Neurological diseases	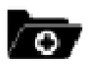	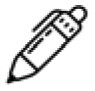	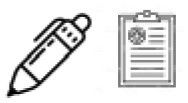	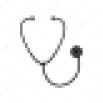
Gastrointestinal diseases	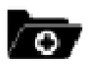	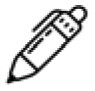	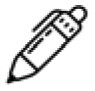	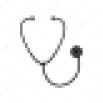
Hearing/vision	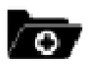	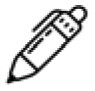	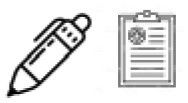	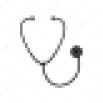
Hospitalizations	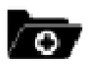	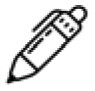	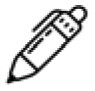	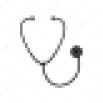
Sleep	–	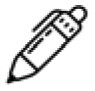	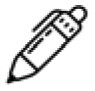	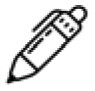
Breastfeeding	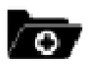	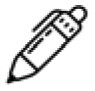	–	–
Eating behaviour	–	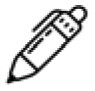	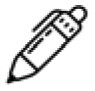	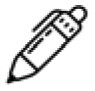
Development and behaviour				
Global development (ASQ)	–	–	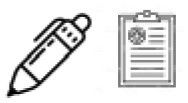	–
Language skills (IFDC, WPPSI IV)	–	–	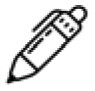	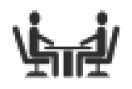
Full-scale Intelligence Quotient (WPPSI IV)	–	–	–	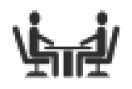
Executive functions (NEPSY 2)	–	–	–	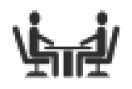
Behaviour and autism spectrum disorders	–	–	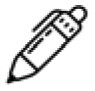	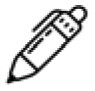
SDQ	–	–	–	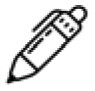
M-CHAT	–	–	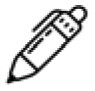	–
SCQ	–	–	–	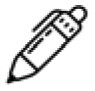
Motor skills	–	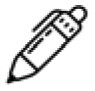	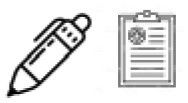	–
M-ABC2	–	–	–	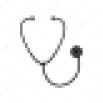
Cerebral palsy^b^	–	–	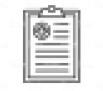	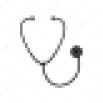
Health care use^c^	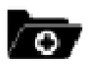	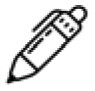	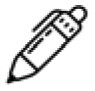	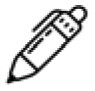
Medications	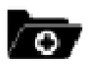	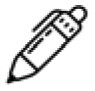	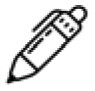	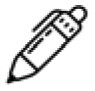
Vaccinations	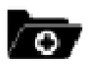	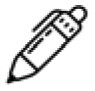	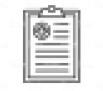	–
Quality of life (PedsQL™)	–	–	–	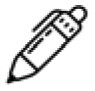
Child care and school attendance	–	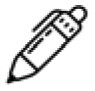	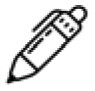	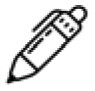

The table specifies whether the information was collected from medical records (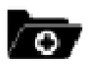), parental self-administered questionnaire (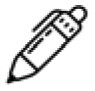), medical questionnaire (
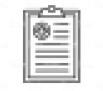
), medical examination (
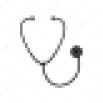
), neuropsychological assessment (
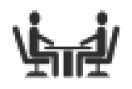
) or not collected at this follow-up (-).

ASQ, Ages and Stages Questionnaire; IFDC, French Communicative Development Inventories (inventaires français du développement communicative, adapted from the MacArthur CDI—communicative development inventories); M-ABC2, Movement Assessment Battery for Children, 2nd edition; M-CHAT, Modified Checklist for Autism in Toddlers^18^; NEPSY 2, Developmental NEuroPSYchological Assessment, 2nd edition; PedsQL™, Pediatric Quality of life Inventory; SCQ, Social Communication Questionnaire; SDQ, Strengths and Difficulties Questionnaire; WPPSI-IV, Wechsler Preschool and Primary Scale of Intelligence, Fourth Edition.

a Gestational age was estimated by obstetric teams, as part of routine care, based on the best obstetric estimate combining last menstrual period and early ultrasonography assessment.

b Diagnosed according to the diagnostic criteria proposed by the Surveillance of Cerebral Palsy in Europe (SCPE) network.^19^ Functional abilities were investigated by using the Gross Motor Function Classification System (GMFCS)0.^20^

c Including hospital admission.

**Table 5:** Standardized scales used in the EPIPAGE-2 questionnaires

	Sweeps	Completeness	Validation in French
Maternal health			
Center for Epidemiologic Studies Depression Scale (CES-D)^21^	Birth	Full version	Yes
State-Trait Anxiety Inventory for adults (STAI)^22^	Birth	Full version	Yes
36-items Short-Form Survey (SF-36)^23^	Birth	Partial	Yes
	1 year	Partial/12 items	Yes
	2 years	Partial/6 items	Yes
	5.5 years	Partial/11 items	Yes
Child’s health			
Ages and Stages Questionnaire (ASQ)^24^	2 years	Full version	Yes
MacArthur-Bates Communication Development Inventories (CDI)^25^	2 years	Full version	Yes
Modified Checklist for Autism in Toddlers (M-CHAT)^26^	2 years	Full version	Yes
Wechsler Preschool and Primary Scale of Intelligence, 4th edition (WPPSI-IV)	5.5 years	Full version	Yes
NEuroPSYchological assessment, 2nd edition (NEPSY 2)^27^	5.5 years	8 subtests^a^	Yes
Strengths and Difficulties Questionnaire (SDQ)^28^	5.5 years	Full version	Yes
Social Communication Questionnaire (SCQ)^29^	5.5 years	Full version	Yes
Movement Assessment Battery for Children, 2nd edition (MABC-2)^30^	5.5 years	Full version	Yes
Children's Eating Behaviour Questionnaire (CEBQ)^31^	5.5 years	Partial/19 items	No
Pediatric Quality of Life Inventory^32^	5.5 years	Partial/23 items	Yes
Home Observation for Measurement of the Environment (HOME) inventory-Short Form	5.5 years	Partial/23 items	Yes

a Inhibition, statue, phonological processing, speed naming, comprehension of instructions, effect recognition, theory of mind, visuomotor precision.

### Unit policies and practices

Another part of the EPIPAGE-2 study focused on the policies and practices of maternity and neonatal units. In 2012, questionnaires were sent to the medical teams of maternity and neonatal units to collect data on their structural characteristics, organization, and policies and practices related to medical interventions and decision-making processes. In total, 98% and 90% of type III and II maternity units and 100% and 98% of type III and II neonatal units, respectively, completed the questionnaire.

### Linkage to routine data sources

Linkage with the national health insurance fund reimbursement register (SNIIRAM) at the individual level is ongoing for families who did not express opposition. It will provide information on prescribed medications since birth and visits to medical and other health care professionals, as well as hospital admissions and their causes. Similar data will be retrieved for the mother during pregnancy. Notably, the linkage will allow for passive follow-up of the children lost to follow-up as long as their parents have not explicitly asked to withdraw from the study.

### Additional projects

The EPIPAGE-2 cohort has also allowed for setting up nine associated projects and two randomized controlled trials (Table 6). Benefiting from the cohort infrastructure, these projects were designed to test very specific associations or interventions in various areas. Accordingly, additional clinical and imaging data as well as biological samples have been collected (Table 6).

**Table 6:** Additional projects nested in the EPIPAGE-2 cohort

Projects	Objectives/number of included children	Funding	Age at material/data collection	Collected material/data
CHORHIST	Histological chorioamnionitis and subsequent health outcomes N = 1406	EQUIPEX—ANR-11-EQPX-0038	Birth	Histological data on placentas
EPIPPAIN 2	Painful procedures in NICU and subsequent neurodevelopment N = 562	Fondation CNP and Regional Hospital Clinical Research Program (PHRC), 2011	Birth	Data on painful procedures in level-III neonatal care units
OLIMPE	Early mother-infant interactions and attachment and subsequent development *N* = 167	Fondation de France, 2011	Birth, 6 months	Data on mother-infant attachment
ETHICS	Antenatal and postnatal decision-making processes regarding extremely preterm infants *N* = 419	Fondation de France, 2010	Birth	Data on limitations of care
EPIRMEX	Cerebral lesions detected by magnetic resonance imaging and development *N* = 313	National Hospital Clinical Research Program (PHRC) 2011	Birth	Data from magnetic resonance imaging (*n *= 298)
EPINUTRI	Neonatal nutrient intake and child development *N* = 325	National Hospital Clinical Research Program (PHRC) 2013	Birth	Data on infant’s polyunsaturated fatty acids and iron intake
			1 year 3 years	Data on child’s diet
EPIFLORE	Intestinal microbiota and diseases of early childhood, childhood and adolescence *N *= 729	ANR 2013	Birth	Infant stools (*n* = 720)
			3 years	Child stools (*n* = 212)
BIOPAG	Biological markers and short- and long-term complications in children *N* = 163	EQUIPEX—ANR-11-EQPX-0038	Birth	Maternal blood (DNA, *n* = 148; RNA, *n *= 147) Cord blood (DNA, *n *= 163; RNA, *n* = 150)
EPIPAGE-2		EQUIPEX—ANR-11-EQPX-0038	5.5 years	Saliva (*n* = 1335)
EPIVAREC	Influence of early nutritional practices in neonatology on children’s ‘metabolic’ status at 5.5 years and its link with growth trajectories *N* = 401	Nestlé	5.5 years	Child’s urine (*n* = 175) Data on body composition, pulse wave velocity, presence of micro-albuminuria ActiGraph-collected data
EPILANG	Randomized controlled trial of a speech-language guidance program *N *= 52	ANR-13-APPR-0007 and National Hospital Clinical Research Program (PHRC) 2013	2 years 5.5 years	Language score of the Developmental Neuropsychological Assessment (NEPSY)
EPIREMED	Randomized controlled trial of cognitive training on visuospatial processing *N* = 170	National Hospital Clinical Research Program (PHRC) 2015	5 years 7 years	Primary index scores of the Wechsler Preschool and Primary Scale of Intelligence (WPPSI IV)

## What has it found?

More than 50 articles based on EPIPAGE-2 data were published up to November 2020, including in collaboration with other cohorts. Details and updates of scientific publications can be found on the EPIPAGE-2 website [https://epipage2.inserm.fr/index.php/en/related-research/scientific-publications]. Some key results are summarized below.

### Short- and mid-term health outcomes

Along with providing up-to-date estimates of health outcomes of preterm children, we have shown substantial improvements in both survival and survival without severe morbidity at discharge for newborns born at 25–31 weeks in 2011 compared with 1997.^33^ There was also an increased use of evidence-based practices known to be beneficial for the newborn (antenatal corticosteroids, surfactant etc.).^33^ These findings were confirmed at 2 years’ corrected age, with a significant increase in survival without severe or moderate neuromotor or sensory disabilities in 2011 compared with 1997.^34^ However, a high number of very and moderately preterm children remained at risk of developmental delay at 2 years of age, which underlines the need for formal developmental evaluations.^34^ The use of standardized parental assessments [Ages and Stages Questionnaire (ASQ), communicative development inventories (CDI)] was considered a valuable screening approach to allow referral of children to a professional if they might benefit from early interventions.^34^^,^^35^ However, this screening strategy will have to be validated with outcomes and specific needs at later stages.

### Extreme prematurity (22–26 weeks)

Survival of extremely preterm children in France was lower than in several other developed countries because of less active antenatal and postnatal care.^33^^,^^36–39^ Moreover, infants born in type III hospitals with higher intensity of perinatal care showed improved survival at 2 years’ corrected age, with no increase in sensorimotor morbidity.^40^ Accordingly, French practices were reassessed and new recommendations were issued in 2020 by French medical associations.

### Obstetric determinants of preterm children’s prognosis

Another major contribution of the EPIPAGE-2 cohort study has been to further study antenatal and obstetric predictors of child outcomes. We developed a new clinically relevant classification of causes of preterm birth,^41^ which was used to more accurately describe preterm newborns’ and children’s prognosis.^42–44^ Other studies have focused on specific pregnancy complications, their management and related health outcomes.^45^^,^^46^

### Evaluation of medical interventions, unit policies and organization of care

EPIPAGE-2 gave us the opportunity to evaluate a large variety of non-consensual or controversial medical interventions and practices in a real-life setting. We have shown that tocolysis administration after preterm premature rupture of membranes (PPROM), although frequently used, was not associated with improved outcomes.^47^ In addition, planned cesarean section was not associated with improved neonatal and 2-year outcomes for preterm twins or preterm cephalic or breech singletons born after preterm labour or PPROM.^48–50^ The comparison of antenatal and postnatal assessments of fetal growth restriction revealed discordances for 14% of very preterm infants, birthweight being more relevant for identifying infants with increased risk.^51^

For infants born before 29 weeks, we showed that echocardiography screening before Day 3 of life was associated with lower in-hospital mortality,^52^ that treating isolated hypotension was associated with improved short-term outcomes^53^ and that early extubation was not associated with an increased risk of intraventricular haemorrhage.^54^

A slow progression of enteral feeding and a less favourable direct-breastfeeding unit policy, as well as some specific microbiota patterns, were associated with the development of necrotizing enterocolitis.^55^ There were large variations in breastfeeding at discharge, regardless of individual factors, which were partly explained by unit policies, suggesting that improvements in unit policies could result in increasing breastfeeding rates.^56^^,^^57^

Neurodevelopmental care implementation is advocated by parent associations. We investigated its dissemination in French neonatal intensive care units (NICUs), showing the essential role of unit policies and the beneficial impact of structured programmes, such as the Newborn Individualized Developmental Care and Assessment Program (NIDCAP), on this dissemination.^58^^,^^59^

We also explored the regionalization of care, showing lower NICU volume associated with lower survival, with no difference in disabilities at 2 years.^60^

### Collaborations

Besides being a very federative project for French clinicians and researchers, the large array of clinical data and biological material collected in the EPIPAGE-2 cohort has led to a number of national and international collaborations.

At the national level, EPIPAGE-2 is closely associated with the ELFE birth cohort [https://www.elfe-france.fr/], whose 18 000 children born at term or near term in France in 2011 serve as a comparison group for some research questions, owing to the collection of similar data.^61^ These two cohorts led to the creation of the RE-CO-NAI research platform, which provides researchers with a database for 22 500 children.

EPIPAGE-2 is part of three projects conducted within the European Union’s Seventh Framework and Horizon 2020 research and innovation programmes: EPICE (Effective Perinatal Intensive Care in Europe, [https://www.epiceproject.eu],^62^ SHIPS (Screening to Improve Health in Preterm Infants in Europe), and RECAP-preterm (Research on European Children and Adults born Preterm, [https://recap-preterm.eu/]). International comparisons of practices and outcomes were also initiated.^63^

The variability of practices and health outcomes described in EPIPAGE-2 has led to setting up multidisciplinary working groups, gathering stakeholders from the French perinatal community and parent associations, aiming at fostering strategies at the national level regarding the perinatal management of extremely preterm babies or the dissemination of neurodevelopmental care. Findings were also used to update French guidelines for clinical practice.^64^^,^^65^

## What are the main strengths and weaknesses?

Strengths include the large size of the cohort, the population-based design at a national level and the prospective enrolment and longitudinal follow-up of infants born preterm. To the best of our knowledge, there is no comparable study covering a broad spectrum of preterm infants from the limits of viability to moderate prematurity. This multidisciplinary project offers an extensive variety of data, enriched by the use of standardized definitions and measures and the collection of biological samples, imaging data and parents’ perceptions of their children’s care and development. The use of standardized tools at the national level to assess development at 5.5 years contributes to harmonizing practices and increasing the quality of evaluations in everyday practice. Moreover, individual-level outcomes and unity policies can be studied simultaneously, in a public health approach.

However, because of its observational nature, establishing causation is sometimes difficult, which is mitigated by the use of adapted statistical methods. Refusal at baseline and attrition over time can lead to selection bias, although families have demonstrated their commitment to collaborating with us. We found a social bias in participation, at baseline and over the years, leading to an under-representation of children from families with disadvantaged socioeconomic positions, as commonly described in other cohorts.^61^ This will hopefully be mitigated by passive data collection through linkage with data from the national health insurance fund reimbursement register. Unfortunately, only few data were collected about fathers and their health. Our sample size is sometimes a limitation to studying rare diseases, which reinforces the need to pool data with other cohort studies. Finally, the costs and organizational challenges are a major obstacle to the cohort’s sustainability, although following these families until adulthood would be of invaluable interest.

## Can I get hold of the data? Where can I find out more?

EPIPAGE-2 was conceived as a research platform to serve the national and international scientific community, with an open data access policy under conditions that ensure data security and confidentiality. To date, data have been requested for 117 projects from 17 different French research institutes or universities and two international projects. Our longitudinal dataset has great potential for collaborations and other secondary analyses. We therefore welcome proposals for data access. The data are accessible to all research teams, French or foreign. The study protocol and the data access procedure can be found on the EPIPAGE-2 website [https://epipage2.inserm.fr/index.php/en/related-research/access-to-epipage-2-data]. Questionnaires and data catalogues are available on the Pandora platform [https://pandora-epipage2.inserm.fr/public/]. All requests are evaluated by the EPIPAGE-2 Data Access Committee and approved on the basis of scientific quality. A contract is signed for each data sharing. Data are usually transferred to French teams within 1 month of the proposal submission. This time frame may be longer for international teams due to the need to establish data-sharing agreements. A specific procedure for ELFE—EPIPAGE-2 joint projects is described at [https://epipage2.inserm.fr/index.php/en/related-research/access-to-epipage-2-data]. Further enquiries should be submitted to Prof. Ancel, contact e-mail: [accesdonnees.epipage@inserm.fr].

**Key Features**

- Etude Epidémiologique sur les Petits Ages Gestationnels-2 (EPIPAGE-2) is a population-based birth cohort of extremely, very and moderately preterm infants, aiming at estimating short- and long-term outcomes and their association with individual characteristics and unit practices.
- Preterm births (terminations of pregnancy, stillbirths and live births) from 22 + 0 to 34 + 6 weeks’ gestation, and occurring in all maternity units of 25/26 regions in France in 2011, were eligible. A total of 7804 newborns were included at baseline (participation rate 93%), and 4312 were eligible for follow-up.
- From 2011 to 2017, three follow-up steps have been performed: at 1-year corrected age (parental self-administered questionnaire, participation 90%) and 2-year corrected age (parental self-administered questionnaire, 88%, medical questionnaire, 86%). At 5.5 years, 3032 children were still followed; the evaluation consisted of a parental questionnaire (77%), a standardized medical examination (68%) and a neuropsychological assessment (67%).
- Detailed information was collected on maternal sociodemographic characteristics, living conditions, health and pregnancy management and complications. Regarding the child, the main domains assessed were health, health care use, nutrition and growth, gross and fine motor skills, cognitive functions, language, behaviour, quality of life and school attendance. Additional data on policies and practices of maternity and neonatal units were also collected.
- Proposals for collaborations and secondary analyses are welcomed. Data access procedures can be found on the EPIPAGE-2 website [https://epipage2.inserm.fr/index.php/en/related-research/access-to-epipage-2-data].

## Funding

The French National Institute of Public Health Research (IRESP TGIR 2009–01 programme)/Institute of Public Health and its partners [the French Health Ministry, the National Institute of Health and Medical Research (INSERM), the National Institute of Cancer, the National Solidarity Fund for Autonomy (CNSA)], the National Research Agency through the French EQUIPEX programme of investments for the future (grant no. ANR-11-EQPX-0038) and the PremUp Foundation. Additional funding was obtained from the Fondation pour la Recherche Medicale (SPF 20160936356) and Fondation de France [00050329 and R18202KK (Grand Prix)].
